# Association of GWAS-Identified Lung Cancer Susceptibility Loci with Survival Length in Patients with Small-Cell Lung Cancer Treated with Platinum-Based Chemotherapy

**DOI:** 10.1371/journal.pone.0113574

**Published:** 2014-11-21

**Authors:** Dong Li, Lixuan Wei, Binghe Xu, Dianke Yu, Jiang Chang, Peng Yuan, Zhongli Du, Wen Tan, Hongbing Shen, Tangchun Wu, Chen Wu, Dongxin Lin

**Affiliations:** 1 State Key Laboratory of Molecular Oncology, Chinese Academy of Medical Science and Peking Union Medical College, Beijing, China; 2 Department of Etiology and Carcinogenesis, Cancer Institute and Hospital, Chinese Academy of Medical Science and Peking Union Medical College, Beijing, China; 3 Department of Medical Oncology, Cancer Institute and Hospital, Chinese Academy of Medical Science and Peking Union Medical College, Beijing, China; 4 Department of Epidemiology and Biostatistics, Cancer Center, Nanjing Medical University, Nanjing, China; 5 School of Public Health, Tongji Medical College, Huazhong University of Science and Technology, Wuhan, China; Duke Cancer Institute, United States of America

## Abstract

Genetic variants have been shown to affect length of survival in cancer patients. This study explored the association between lung cancer susceptibility loci tagged by single-nucleotide polymorphisms (SNPs) identified in the genome-wide association studies and length of survival in small-cell lung cancer (SCLC). Eighteen SNPs were genotyped among 874 SCLC patients and Cox proportional hazards regression was used to examine the effects of genotype on survival length under an additive model with age, sex, smoking status and clinical stage as covariates. We identified 3 loci, 20q13.2 (rs4809957G >A), 22q12.2 (rs36600C >T) and 5p15.33 (rs401681C >T), significantly associated with the survival time of SCLC patients. The adjusted hazard ratio (HR) for patients with the rs4809957 GA or AA genotype was 0.80 (95% CI, 0.66–0.96; *P* = 0.0187) and 0.73 (95% CI, 0.55–0.96; *P* = 0.0263) compared with the GG genotype. Using the dominant model, the adjusted HR for patients carrying at least one T allele at rs36600 or rs401681 was 0.78 (95% CI, 0.63–0.96; *P* = 0.0199) and 1.29 (95% CI, 1.08–1.55; *P* = 0.0047), respectively, compared with the CC genotype. Stratification analyses showed that the significant associations of these 3 loci were only seen in smokers and male patients. The rs4809957 SNP was only significantly associated with length of survival of patients with extensive-stage but not limited-stage tumor. These results suggest that some of the lung cancer susceptibility loci might also affect the prognosis of SCLC.

## Introduction

Lung cancer is the leading cause of cancer deaths all over the world, and categorized into non-small cell lung cancer and small-cell lung cancer (SCLC) [1]. SCLC, accounting for 15%–20% of total lung cancer, is a type of very aggressive neuroendocrine malignancies characterized by high growth rate, widespread metastases and poor prognosis [2], [3]. However, length of survival in patients with SCLC varies greatly and this has been known to be influenced by several clinical factors, such as patient's age, performance status and clinical stage. In recent years, evidence has been accumulated to show that genetic variants might also play a role in the prognosis and length of survival in patients [4], [5]. The identification of such loci might have valuable implication in precision treatment of cancer.

We have previously conducted a genome-wide association study (GWAS) on SCLC to identify genetic variants influencing length of survival in patients and found that the rs1820453T > G SNP, located in the promoter region of the *YAP1* gene which creates a transcription factor binding site and results in down-regulation of *YAP1* expression, is significantly associated [4]. However, this previous GWAS included only 245 samples in the discovery stage and 305 samples in the replication stage and the limited discovery power might obstruct to find most loci with small or moderate effect. Another GWAS on SNPs and survival in NSCLC identified two SNPs, rs7629386 and rs3850370, associated with survival in NSCLC patients derived from both Chinese and Caucasian populations [6]. Thus, other study strategies are warranted to uncover more genetic variants that are associated with length of survival in patients only with SCLC.

In recent years, several GWAS conducted in different ethnic populations to discover susceptibility variants for overall lung cancer have been reported. These published studies have identified at least 26 loci in 13 chromosomal regions that are significantly associated with risk for the development of lung cancer [7]–[15]. In these GWAS, most case subjects were non-small cell lung cancer patients with a proportion of them being patients with SCLC. It has been suggested in many studies that some cancer susceptibility variants may also contribute to disease progression and prognosis [16], [17]. Based on these observations, we sought to examine the hypothesis that the GWAS-identified lung cancer susceptibility loci may also be associated with outcome of SCLC.

Here, we report our study on the association between GWAS-identified lung cancer susceptibility loci and length of survival of SCLC, in which 18 susceptibility loci were analyzed in total of 874 patients. We found that three of these susceptibility loci, 20q13.2 (rs4809957), 22q12.2 (rs36600) and 5p15.33 (rs401681), are significantly associated with length of survival in SCLC patients.

## Materials and Methods

### Ethics statement

All participants provided written informed consent and the ethical committees of Cancer hospital of Chinese Academy of Medical Science and Nanjing Medical University approved this research project.

### Patients and clinical characteristics

A total of 874 patients with SCLC were included in this study. Among them, 569 were recruited at Cancer hospital, Chinese Academy of Medical Science (Beijing) between July 2000 and October 2011 and 305 were recruited at Cancer Hospital of Jiangsu Province, the First Affiliated Hospital of Nanjing Medical University and Nanjing Thoracic Hospital (Nanjing), and four tertiary referral hospitals at Wuhan city, Hubei Province between March 2002 and March 2008. All of them were self-reported ethnic Han Chinese. To be included in this study, all patients had to have cytologically confirmed SCLC and received the first-line carboplatin (AUC 5–6, day 1) or cisplatin (60–80 mg/M^2^, day 1) plus etoposide (100 mg/M^2^, days 1–3) chemotherapy for at least two cycles. Participants did not receive other therapeutics. According to the Veterans' Administration Lung Study Group, patients were classified as having limited disease or extensive disease on the basis of the results of a physical examination; computed tomography scan of the chest, liver, and adrenal glands; a magnetic resonance imaging scan or computed tomography scan of the head; and a bone scan. Characteristics and clinical information including age, sex, smoking status and clinical stage, were obtained from patients' medical records and are shown in **Table 1**. Length of survival of patients was measured from the date of treatment to the date of last follow-up or death. Whether and when a patient had died were obtained from inpatient and outpatient records, patients' families, or local Public Security Census Register Office through follow-up telephone calls. The last date of follow-up was December 20th, 2012. Patients alive on the last follow-up date were considered censored. Written informed consents were from all patients and this study was approved by the Institutional Review Board of Cancer hospital, China Academy of Medical Science. Most patients have been reported in our previous study [4].

**Table 1:** Clinical characteristics of 874 patients with small-cell lung cancer.

Characteristics	*N* = 874		
	No. (%)	MST (month)	*P* †
Dead	521 (59.6)	25	
Alive	353 (40.4)		
Sex			0.0274
Male	666 (76.2)	24	
Female	208 (23.8)	29	
Age			0.0018
≤50 years	231 (26.4)	27	
51–60 years	318 (36.4)	27	
>60 years	325 (37.2)	22	
Smoking status			0.0229
Nonsmoker	249 (28.5)	29	
Smoker	625 (71.5)	24	
Clinical stage*			<0.0001
Limited	479 (54.8)	32	
Extensive	395 (45.2)	18	

Abbreviation: No., number of patients; MST, median survival time.

^†^ *P* values for log-rank test.

*Classified according to the Veterans' Administration Lung Study Group.

### SNP selection, genotyping and quality control

Genomic DNA from each patient was extracted from blood samples using commercial Flexi Gene DNA extraction kit (Qiagen, Hilden, Germany). Twenty-six SNPs at 13 chromosomal regions were reported to be associated with risk of lung cancer in the previous GWAS [7]–[15]. We did quality control of these SNPs using the genotyping information from Version 3 of 1000 Genomes Project data. Among these SNPs, six SNPs with minor allele frequency (MAF) <0.05 were excluded. We then computed the correlation coefficient (r) of each pair of adjacent SNPs at the same chromosome to assess the LD status. SNPs with r^2^>0.8 were considered to be in one LD block, and we thus selected one SNP in the block for further analyses. With these criteria, we finally selected 18 tagSNPs for genotyping in this study. The information of these loci was shown in **Table 2**. Among these loci, only 15 can be readily genotyped by using the MassARRAY system (Sequenom, San Diego, CA). Two loci, rs17728461 and rs2736100, were genotyped by TaqMan assays using ABI 7900HT system (Applied Biosystems, Foster City, CA). Due to the failure of genotyping using both Sequenom or TaqMan assay, the remaining rs2395185 SNP was replaced with rs28366298 SNP as a surrogate, a locus in perfect linkage disequilibrium (LD) with rs2395185 (r^2^ = 1.00) in the same LD block at 6p21.32 and this SNP was also genotyped by TaqMan assay. The primers and probes for genotyping, which were commercially designed by ABI Company (Applied Biosystems), are available upon request. Several quality-control measures were implemented in genotyping analysis, including (i) duplicated samples were mixed in the plates; (ii) persons performing the genotyping assays were not aware of the status of the duplicated samples; (iii) both positive and negative (no DNA) control samples were included on every 384­well assay plate and (iv) 20% masked random samples were genotyped twice by different investigators and all the results were completely concordant, with the concordance being 100%.

**Table 2:** Associations of 18 candidate SNPs and survival of patients with small-cell lung cancer.

SNP ID	Chromosome	Putative Gene	Minor Allele	MAF	HR (95% CI)†	*P* *
						
**rs4809957**	**20q13.2**	***CYP24A1***	**A**	**0.38**	**0.84 (0.74–0.96)**	**0.0098**
**rs36600**	**22q12.2**	***MTMR3***	**T**	**0.12**	**0.82 (0.68–0.98)**	**0.0261**
**rs401681**	**5p15.33**	***CLPTM1L***	**T**	**0.29**	**1.14 (1.01–1.28)**	**0.0356**
rs17728461	22q12.2	*HORMAD2*	G	0.22	0.87 (0.75–1.01)	0.0594
rs1663689	10p14	*GATA3*	C	0.37	0.89 (0.78–1.01)	0.0634
rs2853677	5p15.33	*TERT*	G	0.39	0.92 (0.81–1.04)	0.1841
rs247008	5q31.1	*CSF2*	A	0.47	0.92 (0.82–1.04)	0.2063
rs28366298	6p21.32	*HLA-DRB1*	C	0.36	1.08 (0.96–1.22)	0.2153
rs10937405	3q28	*TP63*	T	0.32	1.09 (0.95–1.25)	0.2466
rs2736100	5p15.33	*TERT*	C	0.42	0.94 (0.83–1.07)	0.3762
rs753955	13q12.12	*MIPEP*	G	0.37	0.95 (0.83–1.08)	0.4002
rs465498	5p15.33	*CLPTM1L*	G	0.18	1.08 (0.90–1.28)	0.4148
rs2895680	5q32	*STK32A*	C	0.32	0.96 (0.84–1.10)	0.5410
rs7216064	17q24.3	*BPTF*	G	0.36	1.04 (0.92–1.18)	0.5414
rs7086803	10q25.2	*VTL1A*	A	0.26	1.03 (0.89–1.19)	0.6792
rs4488809	3q28	*TP63*	C	0.49	0.98 (0.87–1.11)	0.7392
rs9387478	6q22.2	*DCBLD1*	C	0.48	0.98 (0.87–1.11)	0.7726
rs8042374	15q24	*CHRNA3*	A	0.27	1.01 (0.88–1.15)	0.9128

Abbreviation: MAF, minor allele frequency; HR, hazard ratio; CI, confidence interval. The results with *P*<0.05 were shown in bold.

^†^Calculated with multivariate Cox regression under an additive genetic model adjusted for age, sex, smoking status and clinical stage.

*P values were obtained from the comparisons of the minor allele with the major allele.

### Statistical analysis

For the association between each SNP and length of survival of SCLC patients, we conducted a Cox proportional hazards regression under a log-additive genetic model and hazard ratio (HR) and their 95% confidence interval (CI) were adjusted for age (≤50, 51–60 or >60 years), sex (male or female), smoking status (nonsmoker or smoker) and clinical stage (limited stage or extensive stage). Kaplan-Meier survival estimates were assessed using the log-rank test. All statistical tests were carried out in a two-sided manner using the ‘survival package’ in R.

## Results

### Patient characteristics

The clinical characteristics of 874 SCLC patients are shown in **Table 1**. Up to the last follow-up date, 521 (59.6%) patients had died of SCLC, with a median survival time (MST) of 25 months; 353 (40.4%) patients are still alive. The median follow-up time was 40 months. Among these patients, 479 (54.8%) had limited disease and 395 (45.2%) had extensive disease. The MST for limited disease was 32 months and for extensive disease was 18 months, indicating that clinical stage is a parameter strongly associated with length of survival in SCLC patients (*P*<0.0001). In addition, patient's age was also strongly associated with length of survival, with older patients (>60 years) having shorter survival time than younger patients (≤60 years) (*P* = 0.0018).

### Association of genetic susceptibility loci with length of patients' survival

We found that, among the 18 loci analyzed, three SNPs, i.e., rs4809957 in *CYP24A1* at 20q13.2, rs36600 in *MTMR3* at 22q12.2 and rs401681 in *CLPTM1L* at 5p15.33, were significantly (all *P*<0.05) associated with length of survival in SCLC patients (**Table 2**).

**Figure 1:**
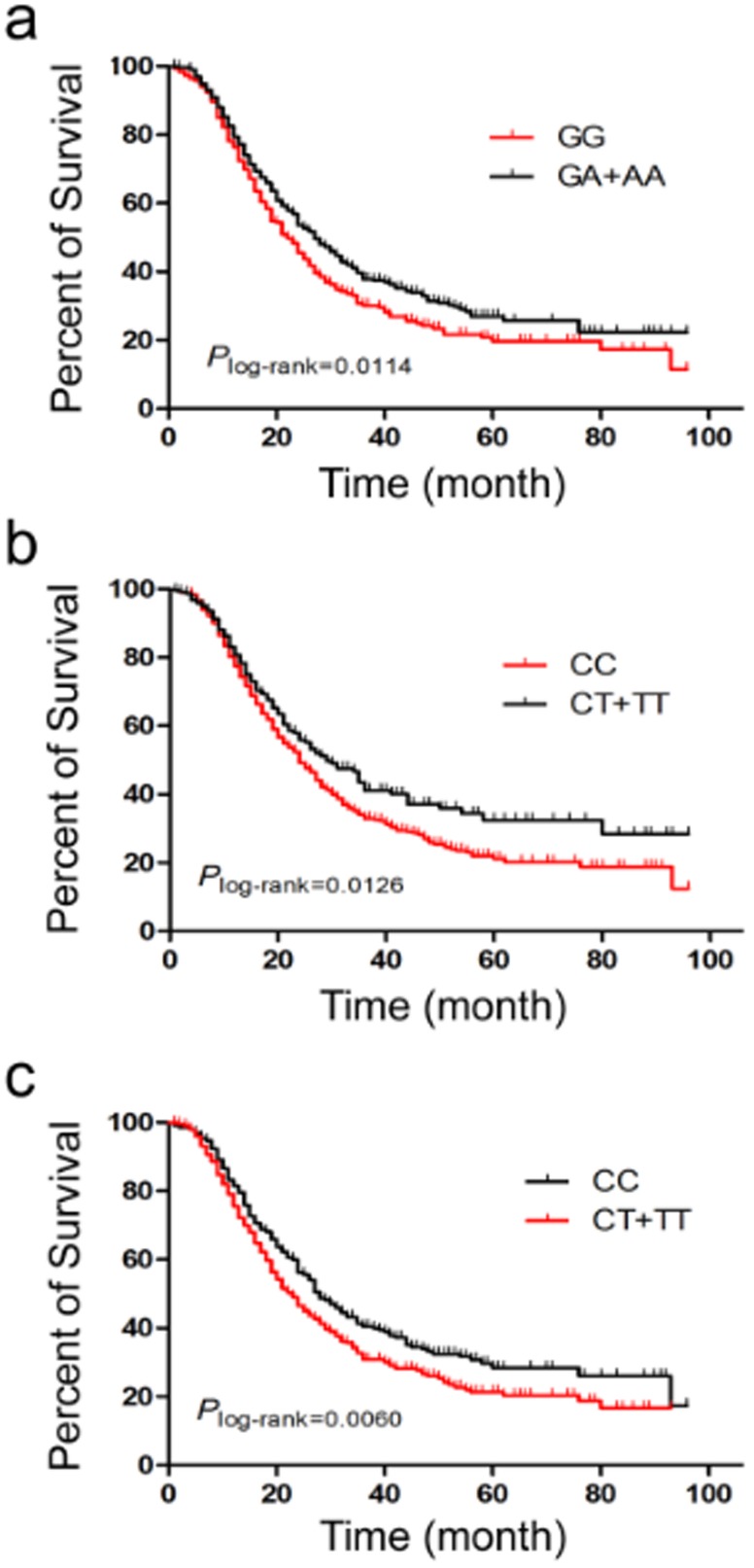
Kaplan–Meier estimates of overall survival of patients with small-cell lung cancer according to rs4809957G >A (a), rs36600C >T (b) or rs401681C >T (c) genotypes.

The rs4809957G >A locus was the most significant one, with the adjusted HR for death of patients being 0.84 (95% CI, 0.74–0.96; *P* = 0.0098) under the additive model (**Table 2**). The MST for the rs4809957 GG, GA or AA genotypes was 22, 27 or 28 months, respectively. The adjusted HR for death of patients with the rs4809957 GA or AA genotype was 0.80 (95% CI, 0.66–0.96; *P* = 0.0187) and 0.73 (95% CI, 0.55–0.96; *P* = 0.0263) compared with the GG genotype (**Table 3**). In a dominant model, patients with the rs4809957 GA or AA genotype had significantly longer MST (27 months) than those with the GG genotype, with the adjusted HR being 0.78 (95% CI, 0.65–0.93; *P* = 0.0067) (**Table 3** and **Fig. 1**).

**Table 3:** HR and MST of patients with small-cell lung cancer for the 3 significant SNPs.

Genotype	No.	Dead/Alive	MST (months)	HR (95% CI)†	*P* *
rs4809957					
GG	299	194/105	22	1.00 (Reference)	
GA	433	250/183	27	0.80 (0.66–0.96)	0.0187
AA	127	67/60	28	0.73 (0.55–0.96)	0.0263
GA+AA	560	317/243	27	0.78 (0.65–0.93)	0.0067
rs36600					
CC	640	396/244	24	1.00 (Reference)	
CT	197	102/95	30	0.78 (0.63–0.98)	0.0296
TT	23	14/9	24	0.77 (0.45–1.31)	0.3336
CT+TT	220	116/104	29	0.78 (0.63–0.96)	0.0199
rs401681					
CC	379	209/170	28	1.00 (Reference)	
CT	360	224/136	23	1.35 (1.11–1.63)	0.0022
TT	115	74/41	24	1.20 (0.92–1.57)	0.1819
CT+TT	475	298/177	23	1.29 (1.08–1.55)	0.0047

Abbreviation: No., number of patients; MST, median survival time; HR, hazard ratio; CI, confidence interval. Because of genotyping failure of some DNA samples, the number of subjects may not add up to the total number.

^†^Calculated with multivariate Cox regression models adjusted for age, sex, smoking status and clinical stage.

*P values were obtained from the comparison with the major genotype.

The rs36600C >T SNP was also significantly associated with length of survival in SCLC patients, with the adjusted HR being 0.82 (95% CI, 0.68–0.98; *P* = 0.0261; Table 2). Compared with patients with the CC genotype (MST, 24 months), patients carrying at least one T allele had longer length of survival (MST, 29 months), with the adjusted HR being 0.78 (95% CI, 0.63–0.96; *P* = 0.0199) (**Table 3** and **Fig. 1**).

In contrast with the above two loci showing favorable effects of minor alleles on patient's survival, the rs401681C >T change showed a poor effect on length of survival, with the adjusted HR for death of patients being 1.14 (95% CI, 1.01–1.28; *P* = 0.0356) under the additive model (**Table 2**). Compared with patients carrying the rs401681 CC genotype (MST, 28 months), patients carrying the CT or TT genotype had significantly shorter survival time (MST, 23 or 24 months) with the adjusted HR being 1.35 (95% CI, 1.11–1.63; *P* = 0.0022) or 1.20 (95% CI, 0.92–1.57; *P* = 0.1819), respectively (**Table 3**).Under a dominant model, patients with at least one T allele had significant shorter survival time (MST, 23 months; adjusted HR, 1.29, 95% CI, 1.08–1.55; *P* = 0.0047) compared with those with the CC genotype (**Table 3** and **Fig. 1**).

Analyses stratified by patients' age, sex, smoking status and clinical stage were further performed and the results are shown in **Table 4**. The association with length of survival of patients for the rs4809957, rs36600 and rs401681 SNPs were only seen in smokers and males. After stratified by clinical stage of the disease, we found that rs4809957 but not rs36600 and rs401681 was specifically significantly associated with length of survival in patients with extensive disease (adjusted HR, 0.80, 95% CI, 0.67–0.96; *P* = 0.0181). The 3 SNPs did not display significantly different association with length of patient survival in terms of patients' age.

**Table 4:** Stratification analysis of association for the 3 significant SNPs.

	rs4809957		rs36600		rs401681	
	HR (95% CI)†	*P* *	HR (95% CI)†	*P* *	HR (95% CI)†	*P* *
Sex						
Male	**0.84 (0.72–0.97)**	**0.0202**	**0.81 (0.66–0.99)**	**0.0387**	**1.15 (1.01–1.31)**	**0.0414**
Female	0.83 (0.63–1.10)	0.1925	0.83 (0.55–1.25)	0.3653	1.05 (0.79–1.42)	0.7246
Age, years						
≤50	0.77 (0.59–1.00)	0.0538	0.75 (0.52–1.09)	0.1344	1.14 (0.90–1.46)	0.2786
51–60	0.80 (0.63–1.01)	0.0645	0.92 (0.67–1.26)	0.5866	1.20 (0.97–1.49)	0.0884
>60	0.93 (0.75–1.14)	0.4693	0.77 (0.59–1.02)	0.0635	1.08 (0.90–1.30)	0.3913
Smoking status						
Nonsmoker	0.83 (0.65–1.08)	0.1613	0.90 (0.62–1.30)	0.5558	1.02 (0.79–1.31)	0.9065
Smoker	**0.85 (0.73–0.99)**	**0.0405**	**0.79 (0.64–0.97)**	**0.0221**	**1.17 (1.02–1.34)**	**0.0279**
Clinical stage						
Limited	0.90 (0.75–1.09)	0.2933	0.78 (0.60–1.02)	0.0702	1.15 (0.96–1.38)	0.1342
Extensive	**0.80 (0.67–0.96)**	**0.0181**	0.86 (0.68–1.10)	0.2331	1.11 (0.94–1.30)	0.2068

Abbreviation: HR, hazard ratio; CI, confidence interval. The results with *P*<0.05 are shown in bold.

^†^Calculated with multivariate Cox regression under an additive genetic model adjusting for age, sex, smoking status and clinical stage where is appropriate.

*P values were obtained from the comparisons of the minor allele with the major allele.

## Discussion

Based on the GWAS-identified lung cancer susceptibility loci, this study explored whether they are also associated with length of survival in SCLC patients. We found that, of the 18 investigated lung cancer susceptibility SNPs, 3 are also associated with survival of Chinese SCLC patients. To the best of our knowledge, this is the first report connecting the lung cancer susceptibility loci to the prognosis of SCLC. Our results are in line with the findings that some cancer susceptibility variants may also contribute to disease progression and prognosis [16], [17]. Our results denoted that male patients were more susceptibility to cancer aggression, as compared with female patients, evidenced by less survival rate. This observation is congruent with recent publications, supporting that there is a disparity between genders, where the male's origin cells also exhibited more susceptibility [18], [19].

The rs4809957 SNP is located in the 3′-untranslated region (3′-UTR) of *CYP24A1* at 20q13.2. It has been well known that SNPs located at 3′-UTR of genes might modulate gene expression by affecting certain microRNA's binding to their transcript. As a result, such a SNP in the 3′-UTR of *CYP24A1* might act through impacting the gene expression level to consequently influence patients' survival. *CYP24A1* plays an important role in vitamin D homeostasis in tissues by catabolizing the active form of vitamin D (1,25-D3), which has anti-proliferative effect in cancer, to inactive calcitroic acid. Previous studies have shown that CYP24A1 is overexpressed in many types of human cancer including lung cancer [20], [21] and overexpression of this enzyme is an independent prognostic maker of survival in patients with lung adenocarcinoma [22]. In our previous lung cancer GWAS, it seems that the rs4809957A allele was the risk allele compared with the G allele [9]. However, in the current study, the A allele was found to be the favorable allele for survival of SCLC patients. This disparity effect of the *CYP24A1* variant in lung cancer susceptibility and SCLC survival is currently unknown. To address this, it would be interesting to analyze the allele-specific expression of *CYP24A1* in normal lung tissues and lung cancer tissues.

The rs36600 SNP is located in the intronic region of the *MTMR3* gene at 22q12.2 and the T allele was associated with better survival in SCLC patients. This association direction is different from that for lung cancer susceptibility, as the previous GWAS reported that the rs36600T allele was associated with increased risk of lung cancer [9]. *MTMR3* encodes myotubularin-related protein-3, which belongs to myotubularin phosphatase gene family [23]. It has been shown that *MTMR3* is involved in cancer cell proliferation, migration and invasion [24]. *MTMR3* is also involved in autophagic activity [25], an important mechanism in the inhibition of tumor growth. The rs36600 SNP is located in the intron of *MTMR3*, which might influence the gene splicing or expression [26]. Therefore, it is plausible that genetic variation in *MTMR3* is associated with SCLC survival, although the function of the rs36600 SNP remains elusive.

Located in the *TERT-CLPTM1L* region at 5p15.33 harboring multiple variants that are associated with susceptibility to many types of human cancer, the variant rs401681T allele is associated with increased risk for death of SCLC in this study. Previous GWAS showed that thers401681T allele is associated with decreased risk of lung, prostate, bladder, cervical and basal cell cancers, but increased risk of pancreatic cancer, melanoma and chronic lymphocytic leukemia [27]–[30]. *CLPTM1L*, also known as cisplatin resistance related gene 9 (*CRR9*), has been found to be overexpressed in human ovarian cancer cells that are resistant to cisplatin-induced apoptosis [31]. Recent study also showed that *CLPTM1L* is overexpressed in lung cancer tissues compared with matched normal lung tissues and its overexpression seems to protect from apoptosis induced by cisplatin [32], [33]. Taken together, these findings suggest that the rs401681 SNP may affect the efficacy of platinum-based chemotherapy, the first-line regime for SCLC, which is consequently associated with poor survival in patients.

The present study has several strengths. First, the sample size was relative larger. We recruited 874 patients with SCLC for analysis, which had suitable statistical power to identify the true association with length of survival. Second, main and simple treatment with platinum-based chemotherapy and relatively shorter survival time of SCLC might minimize the bias of our results by unknown confounding factors and enhanced our ability to find genetic factors associated with survival. Therefore, our results are convincing. However, this study also has some limitations. Although patients with SCLC were recruited from several different hospitals, this study should be considered as a single-center study. Thus, confirmation studies with larger sample size from different ethnic populations are needed. In addition, it would be interesting to elucidate the functional relevance of the variants to get insight into the mechanism underlying the association.

In summary, our studies found that three GWAS identified lung cancer susceptibility loci are also associated with length of survival in SCLC patients treated with platinum-based chemotherapy. It seems that, however, these lung cancer susceptibility loci display different direction in the association with survival of patients, suggesting that the acting mechanism of these variant loci may be different between lung cancer susceptibility and prognosis. Our findings might be valuable in precision treatment of patients with SCLC.
