# Correction: Association of GWAS-Identified Lung Cancer Susceptibility Loci with Survival Length in Patients with Small-Cell Lung Cancer Treated with Platinum-Based Chemotherapy

**DOI:** 10.1371/journal.pone.0118689

**Published:** 2015-03-17

**Authors:** The PLOS ONE Staff

In the Funding section, the grant number from the National High-Tech Research and Development Program of China is listed incorrectly. The correct grant number is: 2012AA02A502.
